# Exploring Treatment Paradigms in Type 2 Diabetes: A Comparison of Independent and Combined Therapeutic Approaches

**DOI:** 10.7759/cureus.108406

**Published:** 2026-05-07

**Authors:** Kumar Sambhav, Ambrish Kumar Batham, Avaniben Sharadbhai Nimavat, Rajendra Singh, Niraj Lodha, Himanshu Jha, Aashish Patel

**Affiliations:** 1 Department of Anatomy, All India Institute of Medical Sciences, Bilaspur, IND; 2 Department of Kayachikitsa (Internal Medicine), Institute of Ayurveda, Major SD Singh University, Farrukhabad, IND; 3 Department of Pathology, Dr. N.D. Desai Faculty of Medical Science and Research, Dharmsinh Desai University, Nadiad, IND; 4 Department of Community Medicine, Mahayogi Guru Gorakhnath AYUSH University, Gorakhpur, IND; 5 Department of Internal Medicine, Lodha Hospital and Research Center, Pali, IND; 6 Department of Family Medicine, Down Town Hospital, Guwahati, IND; 7 Department of Kayachikitsa (Internal Medicine), B.M. Kankanwadi Ayurvedic Mahavidyalaya, Belagavi, IND

**Keywords:** combination therapy, glycemic control, insulin resistance, treatment strategies, type 2 diabetes mellitus

## Abstract

Type 2 diabetes mellitus continues to pose a substantial global health burden due to its rising prevalence and complex metabolic nature. Multifactorial defects, including insulin resistance, β-cell dysfunction, and altered incretin activity, limit the effectiveness of single-target therapies. Current treatment strategies often fail to achieve sustained glycemic control, and delays in treatment intensification persist, highlighting the need for more effective and timely therapeutic approaches. This review evaluates independent and combined therapeutic strategies, focusing on their mechanisms, clinical performance, and applicability in diverse patient populations. A narrative synthesis of recent clinical trials, guidelines, and real-world evidence from 2015 to 2025 was conducted to compare outcomes and identify optimal treatment pathways. Evidence indicates that stepwise approaches remain practical and cost-effective, particularly in early disease stages and resource-limited settings. Early combination strategies demonstrate superior glycemic control, improved durability, and additional cardiovascular and renal benefits. Personalized treatment approaches further enhance outcomes by aligning therapy with individual patient characteristics. This approach underscores the importance of integrating mechanistic understanding with clinical decision-making to enhance therapeutic effectiveness. These findings support a shift toward early, targeted, and individualized interventions to optimize long-term disease management. Integration of combination strategies with patient-centered care models may improve clinical outcomes and reduce complications.

## Introduction and background

Type 2 diabetes mellitus (T2DM) is a major global health challenge, with rapidly increasing prevalence in both developed and developing countries [1]. According to recent estimates from the International Diabetes Federation, approximately 537 million adults were living with diabetes in 2021, with projections reaching 783 million by 2045 [2]. This rise is driven by demographic transitions, urbanization, sedentary lifestyles, and increasing obesity rates. The clinical and economic burden of T2DM is considerable, driven by its chronic progressive nature and wide spectrum of complications [3]. These include microvascular complications such as retinopathy, nephropathy, and neuropathy, as well as macrovascular events including coronary artery disease, stroke, and peripheral vascular disease, all of which contribute to reduced life expectancy, impaired quality of life, and escalating healthcare costs [4].

T2DM is characterized by a complex and multifactorial pathophysiology involving interrelated metabolic defects. Peripheral insulin resistance in skeletal muscle and adipose tissue reduces glucose uptake and utilization, while hepatic insulin resistance increases endogenous glucose production, exacerbating hyperglycemia [5,6]. Progressive pancreatic β-cell dysfunction further impairs insulin secretion relative to metabolic demand. Additional contributing mechanisms include impaired incretin effect, dysregulated glucagon secretion, increased renal glucose reabsorption, and chronic low-grade inflammation, collectively sustaining metabolic dysregulation [7]. This multifaceted pathogenesis limits the effectiveness of single-target therapies and provides a strong mechanistic rationale for therapeutic strategies that simultaneously address multiple defects.

The management of T2DM remains suboptimal due to its progressive course and significant interindividual variability [8]. Despite the availability of multiple pharmacological agents, a substantial proportion of patients fail to achieve adequate glycemic control. Clinical inertia, defined as delayed treatment intensification despite suboptimal glycemic levels, continues to be a major barrier [9]. Furthermore, heterogeneity in patient characteristics, including age, disease duration, comorbidities, and socioeconomic status, complicates therapeutic decision-making [10]. Safety considerations such as hypoglycemia risk, weight changes, and drug-specific adverse effects must be balanced against therapeutic efficacy. In addition, disparities in access to newer agents with proven cardiovascular and renal benefits remain a critical issue, particularly in resource-limited settings [11].

T2DM pharmacological management broadly follows two therapeutic paradigms: monotherapy (stepwise escalation) and combination therapy [2]. The independent approach involves initiating treatment with a single agent, most commonly metformin, followed by sequential addition of therapies as glycemic control deteriorates. Metformin is widely regarded as the first-line drug of choice due to its ability to reduce hepatic glucose production, favorable safety profile with minimal risk of hypoglycemia, weight neutrality, cardiovascular benefits, and strong cost-effectiveness profile across diverse healthcare settings [5]. The simplicity, affordability, and ease of implementation of this approach have contributed to its widespread adoption. Pharmacoeconomic analyses, including cost-benefit, cost-minimization, and cost-of-illness studies, further support this strategy by demonstrating reduced direct and long-term healthcare costs associated with early glycemic control and prevention of complications [9]. However, this approach may be insufficient to address the multifactorial nature of T2DM and may delay optimal glycemic control, particularly in patients with high baseline glycemic burden [11].

Combination therapy has emerged as an alternative strategy to address these limitations by targeting multiple pathophysiological pathways simultaneously [12]. These approaches include early initiation of dual or triple therapy, fixed-dose combinations, and injectable regimens integrating complementary mechanisms of action. Although combination therapy offers superior, more rapid glycemic control and improved durability, its clinical adoption must be critically evaluated in the context of higher treatment costs, increased regimen complexity, and potential for cumulative adverse effects. These factors can influence adherence, accessibility, and long-term sustainability of treatment. Consequently, therapeutic decision-making should extend beyond glycemic endpoints to incorporate patient-centered outcomes, particularly quality of life, which is influenced by efficacy, safety, affordability, treatment burden, and patient preferences [1]. In selected populations, combination therapy may also provide additional cardiovascular and renal benefits and may contribute to preservation of β-cell function [13].

Contemporary international guidelines, including those from the American Diabetes Association and the European Association for the Study of Diabetes, emphasize individualized treatment strategies that account for comorbidities, risk profiles, and patient-specific goals [14]. This reflects a transition from a uniform stepwise escalation model toward a more stratified and patient-centered approach [15,16]. Despite these advances, existing literature predominantly evaluates therapeutic strategies based on clinical efficacy, with limited integration of pharmacoeconomic and patient-centered outcomes, particularly in resource-limited settings. This gap highlights the need for a more comprehensive framework to guide therapeutic decision-making.

Objective of the review

This narrative review aims to critically evaluate independent and combination therapeutic approaches in T2DM by integrating evidence across multiple domains, including glycemic efficacy, safety, pharmacoeconomic impact, and patient-centered outcomes such as quality of life. Unlike conventional comparisons that focus primarily on clinical endpoints, this review seeks to provide a multidimensional framework for therapeutic decision-making that aligns treatment strategies with disease characteristics, patient profiles, and healthcare system constraints. The review further explores the role of personalized treatment approaches and contextual applicability in contemporary clinical practice, with an emphasis on optimizing long-term outcomes and cost-effectiveness.

Methodology

This narrative review was conducted to synthesize and critically evaluate existing evidence on independent (stepwise) and combination therapeutic strategies in the management of type 2 diabetes mellitus (T2DM). Although not a systematic review, a structured and transparent approach was adopted to enhance methodological rigor and reproducibility.

Literature Search Strategy

A comprehensive literature search was performed using major electronic databases, including PubMed, Scopus, Web of Science, and Google Scholar. The search covered studies published between 2015 and 2025 to ensure inclusion of recent and relevant evidence. Keywords and Medical Subject Headings terms used in various combinations included “Type 2 Diabetes Mellitus”, “monotherapy”, “combination therapy”, “stepwise treatment”, “glycemic control”, “SGLT2 inhibitors”, “GLP-1 receptor agonists”, “pharmacoeconomics”, “quality of life”, and “treatment outcomes”. Search terms were combined using Boolean operators (AND, OR) to refine retrieval and improve the relevance of results.

An example search strategy used in PubMed was: (“Type 2 Diabetes Mellitus” AND “combination therapy” OR “monotherapy” AND “glycemic control”).

Inclusion and Exclusion Criteria

Studies were included if they met the following criteria: 1) involved adult patients with T2DM; 2) evaluated independent or combination pharmacological therapies; 3) reported outcomes related to glycemic control, safety, cardiovascular or renal effects, cost-effectiveness, or patient-centered outcomes; and 4) were published in peer-reviewed journals in English. Eligible study designs included randomized controlled trials, systematic reviews, meta-analyses, clinical guidelines, and relevant real-world observational studies. Studies focusing on type 1 diabetes, gestational diabetes, pediatric populations, or nonpharmacological interventions alone were excluded.

Study Selection

All retrieved records were initially screened based on titles and abstracts to identify potentially relevant studies. Full-text articles were subsequently assessed for eligibility according to predefined inclusion and exclusion criteria. The selection process was conducted systematically, and any uncertainties regarding study inclusion were resolved through discussion and consensus among the authors.

Data Extraction and Synthesis

Data were systematically extracted from included studies, focusing on study design, population characteristics, intervention type, and key outcomes such as HbA1c reduction, safety profiles, cardiovascular and renal outcomes, cost-effectiveness measures, and patient-centered outcomes including quality of life and adherence.

Extracted data were synthesized narratively, with an emphasis on identifying key themes, comparing outcomes across studies, and highlighting consistencies and discrepancies in findings. Evidence was categorized into domains including glycemic efficacy, safety, metabolic outcomes, cardiovascular and renal benefits, adherence, quality of life, and cost-effectiveness.

Quality and Risk of Bias Assessment

The quality of included studies was assessed qualitatively based on study design, sample size, consistency of findings, and clinical relevance. Greater emphasis was placed on high-quality evidence, particularly randomized controlled trials, systematic reviews, and guideline recommendations. Potential sources of bias, including selection bias, reporting bias, and heterogeneity across study populations and study designs, were considered during evidence synthesis.

Formal risk-of-bias assessment tools were not applied due to the narrative nature of the review; however, studies were critically appraised using established methodological considerations to ensure reliability of the evidence base.

Methodological Considerations

Real-world studies were included to provide context on treatment effectiveness, adherence, and applicability in routine clinical practice. This structured narrative approach allowed integration of diverse evidence sources while maintaining transparency in study selection, evaluation, and synthesis.

## Review

Pathophysiological basis for treatment strategies

Core Defects in T2DM

T2DM is a progressive metabolic disorder characterized by multiple interrelated pathophysiological abnormalities. Insulin resistance is a central defect and occurs in both peripheral tissues and the liver [17]. In skeletal muscle and adipose tissue, impaired insulin signaling reduces glucose uptake and utilization, whereas hepatic insulin resistance increases endogenous glucose production, particularly during fasting [18]. These disturbances collectively contribute to persistent hyperglycemia and metabolic dysregulation.

Another critical component in disease progression is the gradual decline in pancreatic β-cell function. Initially, β-cells compensate for insulin resistance by increasing insulin secretion; however, chronic metabolic stress, glucotoxicity, and lipotoxicity ultimately lead to β-cell dysfunction, exhaustion, and apoptosis [19]. This progressive β-cell failure limits the body’s ability to maintain glycemic homeostasis and necessitates intensification of therapeutic interventions over time [2].

Additional pathophysiological mechanisms further contribute to the complexity of T2DM. Dysregulated glucagon secretion from pancreatic α-cells results in inappropriate hepatic glucose production, particularly in the postprandial state [10]. The incretin system, primarily involving glucagon-like peptide-1 (GLP-1) and glucose-dependent insulinotropic polypeptide, is often impaired, leading to reduced insulin secretion and inadequate glucagon suppression [7]. Moreover, increased renal glucose reabsorption mediated by sodium-glucose cotransporter-2 (SGLT2) contributes to sustained hyperglycemia by limiting urinary glucose excretion [1].

**Figure 1:**
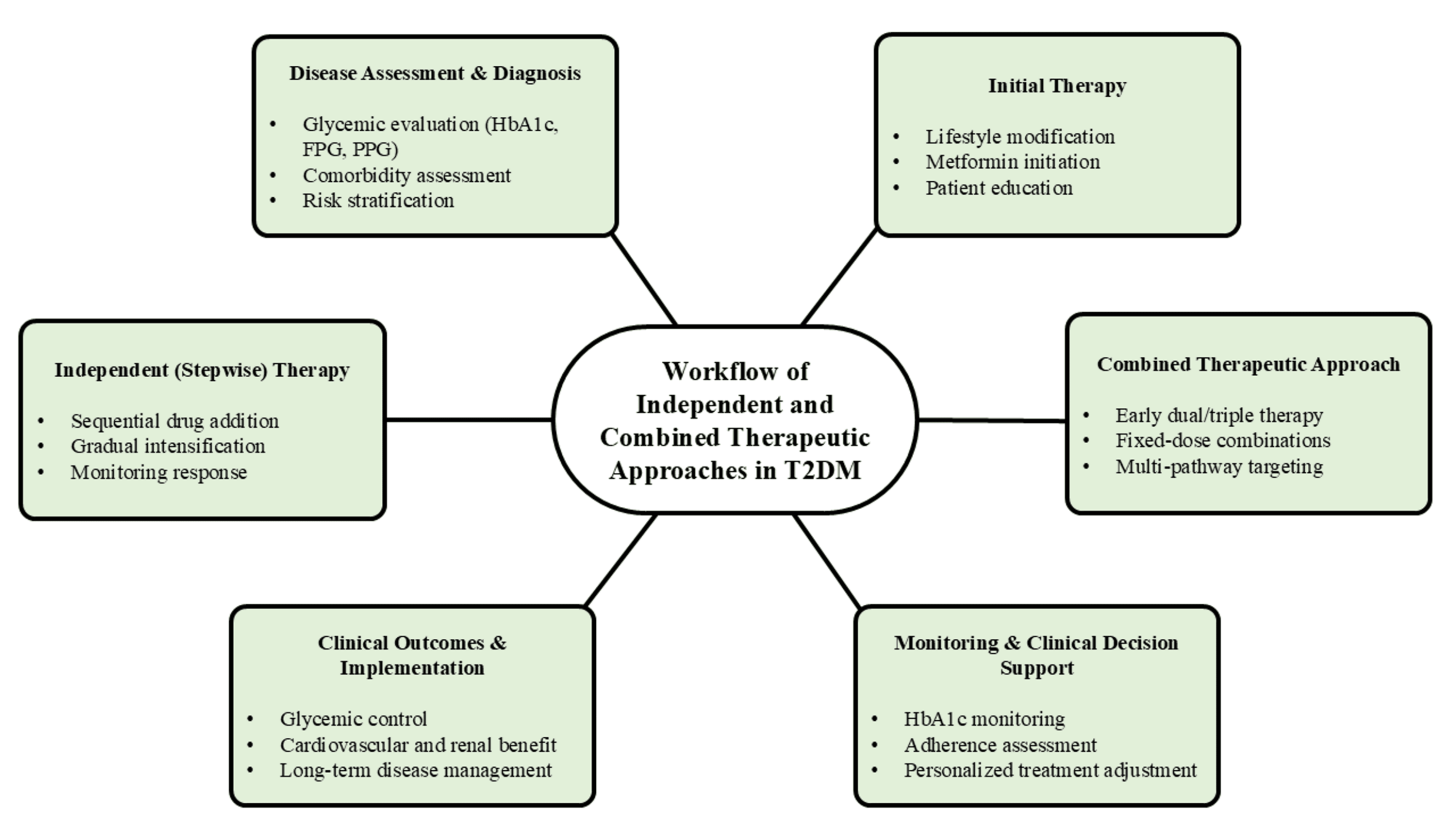
Therapeutic Workflow in T2DM HbA1c: glycated hemoglobin; FPG: fasting plasma glucose; PPG: postprandial glucose; T2DM: type 2 diabetes Image credit: This is an original image created by the author Kumar Sambhav using Microsoft PowerPoint (Microsoft, Redmond, WA)

These interdependent defects highlight the multifactorial nature of T2DM and provide a mechanistic basis for multidimensional therapeutic strategies. Interventions targeting a single pathway may be insufficient, whereas approaches that address multiple underlying defects simultaneously are more likely to achieve durable glycemic control. This pathophysiological framework underpins the rationale for comparing independent and combination therapeutic strategies in T2DM. Figure 1 illustrates the integrated therapeutic workflow in the management of T2DM.

Therapeutic Targets

The primary goal of T2DM management is to achieve and sustain optimal glycemic control, typically assessed using glycated hemoglobin (HbA1c), fasting plasma glucose (FPG), and postprandial glucose (PPG) levels [3]. Effective glycemic control is strongly associated with a reduced risk of microvascular complications and improved long-term clinical outcomes.

Contemporary management strategies extend beyond glycemic targets to encompass broader metabolic and systemic goals. Weight reduction is a key therapeutic objective, particularly in overweight and obese individuals, as it improves insulin sensitivity and overall metabolic status [11]. In addition, mitigation of cardiovascular and renal risk has become a central priority, given the high prevalence of cardiovascular disease and chronic kidney disease among individuals with T2DM [20]. Certain pharmacological agents provide benefits beyond glucose lowering, including cardioprotective and renoprotective effects, which are increasingly influencing treatment selection and prioritization.

These evolving therapeutic objectives necessitate a multidimensional approach to treatment planning. Drug selection is no longer based solely on glycemic efficacy but also incorporates factors such as impact on body weight, risk of hypoglycemia, cardiovascular and renal outcomes, safety profile, and patient-specific characteristics [21]. Furthermore, economic considerations and treatment burden play an important role in real-world decision-making.

This shift toward a comprehensive, patient-centered framework provides the basis for selecting between independent (stepwise) and combination therapeutic strategies. The choice of approach is determined by disease severity, underlying pathophysiological defects, comorbid conditions, and individual patient needs, supporting a more stratified and context-specific model of T2DM management.

Independent therapeutic approaches (monotherapy/stepwise escalation)

Independent therapy in T2DM is based on a stepwise treatment paradigm, in which pharmacological management is initiated with a single agent, typically metformin, and subsequently intensified as glycemic control deteriorates [22]. This approach remains widely adopted in clinical practice due to its structured progression, ease of implementation, and cost-effectiveness, particularly in resource-constrained settings [19].

Within this framework, multiple drug classes are introduced sequentially based on patient response and evolving clinical needs. Sulfonylureas enhance insulin secretion from pancreatic β-cells but are associated with an increased risk of hypoglycemia and weight gain [22]. Dipeptidyl peptidase 4 (DPP-4) inhibitors improve incretin activity and are generally well tolerated with a favorable safety profile. SGLT2 inhibitors (SGLT2i) reduce renal glucose reabsorption and provide additional cardiovascular and renal benefits, while GLP-1 receptor agonists are particularly beneficial in individuals with obesity or high cardiovascular risk [23].

Evidence from clinical trials and long-term observational studies supports the effectiveness of stepwise therapy in achieving glycemic control, particularly in early-stage disease. This approach allows gradual treatment intensification and facilitates identification of drug-specific adverse effects. Its simplicity and lower initial treatment burden are associated with improved early adherence, and pharmacoeconomic analyses indicate that it remains a cost-effective strategy in many healthcare settings [24]. Evidence from clinical trials and long-term observational studies supports the effectiveness of stepwise therapy in achieving glycemic control, particularly in early-stage disease. This approach allows gradual treatment intensification and facilitates identification of drug-specific adverse effects. Its simplicity and lower initial treatment burden are associated with improved early adherence, and evidence from pharmacoeconomic evaluations, including cost-minimization and cost-of-illness analyses, indicates that it remains a cost-effective strategy in many healthcare settings, particularly in resource-constrained environments [2,3].

However, important limitations have been consistently reported. Due to the progressive nature of T2DM, the efficacy of monotherapy declines over time, necessitating treatment escalation [25]. Real-world studies highlight the impact of clinical inertia, with delays in therapy intensification contributing to prolonged hyperglycemia and increased risk of complications. In addition, the sequential targeting of individual pathophysiological defects may be insufficient in a condition characterized by multiple concurrent metabolic abnormalities [15].

Comparative evidence suggests that, although stepwise therapy is effective in early disease, it may be less efficient than early combination strategies in achieving rapid and durable glycemic control. Some studies report comparable short-term outcomes in selected patient groups, but overall findings indicate reduced durability and a higher likelihood of treatment escalation over time [26].

Overall, independent therapy remains a foundational strategy in T2DM management; however, its role is increasingly being reevaluated within modern treatment paradigms that emphasize early intervention, comprehensive metabolic control, and individualized care. The primary elements and clinical implications of independent therapeutic strategies are summarized in Table 1.

**Table 1:** Independent therapy in T2DM T2DM: type 2 diabetes mellitus; DPP-4: dipeptidyl peptidase-4; SGLT2: sodium-glucose cotransporter-2; GLP-1: glucagon-like peptide-1; GRADE: Glycemia Reduction Approaches in Type 2 Diabetes: A Comparative Effectiveness

Domain	Component	Mechanism/description	Clinical implication	Reference
Therapeutic strategy	Stepwise escalation	Initiation with monotherapy (typically metformin) followed by sequential add-on therapy based on glycemic response	Structured, widely applicable, and adaptable treatment pathway	Zaccardi et al. [9]; American Diabetes Association Professional Practice Committee [22]
First-line therapy	Metformin	Reduces hepatic glucose production and improves insulin sensitivity	Effective, safe, weight-neutral, and cost-efficient initial therapy	Grespan et al. [20]
Add-on pharmacological classes	Sulfonylureas	Stimulate insulin secretion from pancreatic β-cells	Effective glycemic control but associated with hypoglycemia and weight gain	Galicia-Garcia et al. [8]
	DPP-4 inhibitors	Enhance incretin activity, increasing insulin secretion and reducing glucagon	Favorable safety profile with low hypoglycemia risk	Green et al. [25]
	SGLT2 inhibitors	Reduce renal glucose reabsorption leading to glycosuria	Provide glycemic control with additional cardiovascular and renal benefits	DeFronzo et al. [21]
	GLP-1 receptor agonists	Improve insulin secretion and reduce appetite	Beneficial in obesity and patients with high cardiovascular risk	Westman [17]
Advantages	-	Simplicity, affordability, and ease of monitoring	Suitable for early-stage disease and resource-limited settings	American Diabetes Association Professional Practice Committee [22]
Limitations	-	Progressive decline in efficacy and clinical inertia	Delayed glycemic control and increased risk of complications	Harding et al. [7]
Evidence base	-	Supported by clinical trials and observational studies	Established role, though increasingly compared with early combination strategies	The GRADE Study Research Group [23]

Commonly used antidiabetic agents and therapeutic combinations in India

In the Indian clinical context, the selection of antidiabetic medications is shaped by multiple interrelated factors, including cost constraints, drug accessibility, patient characteristics, and alignment with national and international clinical guidelines. These considerations are particularly important in a resource-variable healthcare system, where affordability and availability significantly influence therapeutic decisions. A broad spectrum of pharmacological agents is utilized either as monotherapy or in combination to achieve and maintain optimal glycemic control.

Monotherapy options predominantly include biguanides, with metformin remaining the first-line drug of choice due to its efficacy, safety, and cost-effectiveness. Other commonly used agents include sulfonylureas such as glimepiride, glibenclamide, and gliclazide; DPP-4 inhibitors including sitagliptin, vildagliptin, teneligliptin, and linagliptin; SGLT2i such as dapagliflozin, empagliflozin, and canagliflozin; thiazolidinediones such as pioglitazone; GLP-1 receptor agonists including liraglutide, semaglutide, and dulaglutide; and alpha-glucosidase inhibitors such as acarbose and voglibose. The selection among these agents is guided by patient-specific clinical profiles, including glycemic status, comorbidities, and tolerability.

Combination therapy in India is widely practiced and often initiated when glycemic targets are not achieved with monotherapy or in patients presenting with higher baseline glycemic burden. Common dual oral regimens include metformin combined with a sulfonylurea, a DPP-4 inhibitor, an SGLT2i, pioglitazone, or an alpha-glucosidase inhibitor. For further intensification, triple therapy regimens are employed, typically involving metformin in combination with two additional agents targeting complementary mechanisms of action. In advanced or uncontrolled cases, injectable therapies are introduced, including basal insulin in combination with oral agents, GLP-1 receptor agonists combined with basal insulin, and premixed insulin regimens.

A distinctive feature of the Indian pharmaceutical landscape is the widespread availability of fixed-dose combinations, which can reduce pill burden and potentially improve treatment adherence. However, their use must be balanced against considerations of dose flexibility and appropriateness for individual patients. Overall, treatment selection in India reflects a pragmatic, individualized approach that integrates clinical efficacy, safety profile, economic feasibility, and patient preferences, thereby aligning with a broader patient-centered and context-specific model of T2DM management.

Combined therapeutic approaches

Combination therapy in T2DM involves using two or more pharmacological agents, either initiated early in the disease course or introduced during treatment intensification, to achieve more rapid and durable glycemic control [3]. These strategies include early dual or triple therapy, fixed-dose combinations, and injectable regimens such as GLP-1 receptor agonists combined with basal insulin, allowing flexibility across different stages of disease progression [27].

The rationale for combination therapy is strongly supported by the multifactorial pathophysiology of T2DM. Simultaneous targeting of complementary mechanisms, such as insulin resistance, impaired insulin secretion, and increased renal glucose reabsorption, has been shown to enhance therapeutic effectiveness compared with single-agent approaches [21,28]. Commonly employed strategies include oral-oral combinations, for example, metformin with SGLT2i or DPP-4 inhibitors, and oral-injectable regimens in more advanced disease.

Evidence from randomized controlled trials and long-term studies indicates that combination therapy enables faster attainment of glycemic targets and improves durability of glycemic control compared with stepwise escalation [29,30]. Early combination strategies have also been associated with reduced glucotoxicity and potential preservation of pancreatic β-cell function by decreasing metabolic stress. In addition, systematic reviews have demonstrated that certain combinations, particularly those involving SGLT2i or GLP-1 receptor agonists, provide additional benefits, including weight reduction and significant cardiovascular and renal protection, thereby enhancing their clinical relevance in high-risk populations.

However, these advantages must be interpreted in the context of important limitations. Pharmacoeconomic analyses indicate that combination therapy is associated with higher direct treatment costs, which may limit accessibility, particularly in low-resource settings [30]. Increased regimen complexity and the potential for cumulative or drug-specific adverse effects may also impact adherence and long-term sustainability. Real-world evidence suggests that these factors can offset clinical benefits in certain patient groups, highlighting variability in treatment outcomes.

Although most evidence supports the superiority of early combination therapy over conventional stepwise approaches for achieving glycemic control, some studies report comparable outcomes in selected patients with lower baseline disease severity. Furthermore, uncertainties remain regarding optimal drug combinations, long-term safety, and cost-effectiveness across diverse populations [31].

**Figure 2:**
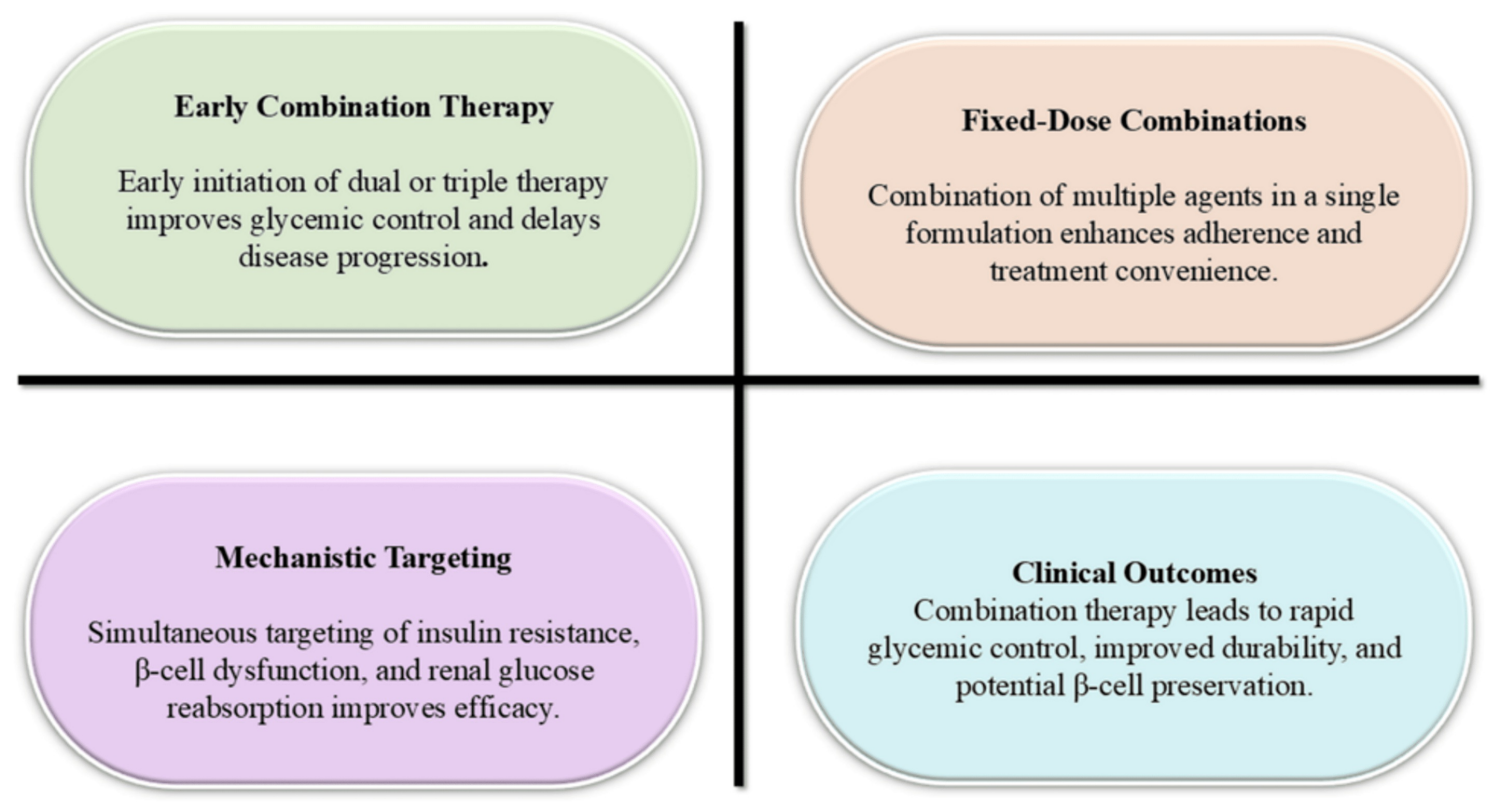
Key components of combined therapeutic approaches in T2DM T2DM: type 2 diabetes mellitus Image credit: This is an original image created by the author Kumar Sambhav using Microsoft PowerPoint (Microsoft, Redmond, WA)

Overall, combination therapy represents a clinically effective strategy within a broader multidimensional treatment framework; however, its application should be individualized based on disease severity, comorbidities, economic considerations, and patient preferences. Figure 2 illustrates the key components of integrated therapeutic strategies in T2DM.

Multidimensional comparative framework: independent vs. combined therapeutic approaches

Framework for Comparison

The comparison between independent and combination therapeutic strategies in T2DM is based on a multidimensional framework integrating glycemic efficacy, safety, metabolic outcomes, cardiovascular and renal benefits, adherence, quality of life, and cost-effectiveness. These parameters are derived from evidence across randomized controlled trials, systematic reviews, clinical guidelines, and real-world studies, providing a structured basis for evaluating therapeutic performance.

Glycemic Efficacy and Durability

Evidence from randomized controlled trials and meta-analyses consistently demonstrates that combination therapy achieves greater and more rapid reductions in HbA1c, FPG, and PPG levels compared with monotherapy or stepwise escalation [5,32]. Several studies also indicate improved durability of glycemic control with early combination therapy [33]. However, some data suggest that short-term outcomes may be comparable in patients with lower baseline glycemic burden, indicating that treatment effectiveness is influenced by disease stage and patient characteristics [6].

Safety and Metabolic Outcomes

Safety profiles vary depending on the pharmacological agents used. Evidence indicates that stepwise approaches frequently involve sulfonylureas or insulin, which are associated with higher risks of hypoglycemia and weight gain [33]. In contrast, combination regimens incorporating SGLT2i or GLP-1 receptor agonists have a lower risk of intrinsic hypoglycemia and are often associated with weight reduction. Systematic reviews further support the cardiovascular and renal benefits of these agents, particularly in high-risk populations [27].

Patient-Centered Outcomes (Adherence and Quality of Life)

Patient-centered outcomes show variable patterns across studies. Simpler monotherapy regimens are associated with improved early adherence; however, real-world evidence suggests that delayed treatment intensification and suboptimal glycemic control may negatively impact long-term treatment satisfaction. Conversely, fixed-dose combination therapies may improve adherence and clinical outcomes by reducing pill burden, although increased regimen complexity and cost remain limiting factors in certain settings [15,30].

Pharmacoeconomic Considerations

From a pharmacoeconomic perspective, cost-minimization and cost-of-illness studies indicate that monotherapy offers lower upfront costs and greater accessibility, particularly in resource-constrained environments [34]. In contrast, long-term analyses suggest that combination therapy may be more cost-effective by reducing complication rates, hospitalization, and the need for repeated treatment escalation [35].

Integrated Interpretation

Overall, while the majority of evidence supports the clinical and metabolic advantages of combination therapy, variability across studies, related to differences in patient populations, baseline disease severity, and healthcare contexts, highlights the need for individualized application. The choice between independent and combination therapy should therefore be guided by a balanced integration of clinical efficacy, safety, economic considerations, and patient-centered outcomes. The major differences are summarized in Table 2.

**Table 2:** Therapy comparison in T2DM T2DM: type 2 diabetes mellitus; SU: sulfonylureas; SGLT2i: sodium-glucose cotransporter-2 inhibitors; GLP-1 RA: glucagon-like peptide-1 receptor agonists

Parameter	Independent approach	Combined approach	Reference
Glycemic control	Gradual reduction	Rapid, greater reduction	Ahmed et al. [32]
Durability	Less durable	More sustained control	Neuen et al. [31]
Hypoglycemia risk	Higher (insulin/SU)	Lower (SGLT2i, GLP-1 RA)	Colombijn et al. [30]
Weight effect	Weight gain	Weight neutral/reduction	Aristizábal-Colorado et al. [34]
Cardio-renal benefit	Limited	Proven benefits	Siddiqui et al. [33]
Adherence	Simple initially	Improved with fixed-dose	Ahmed et al. [32]
Cost	Lower upfront	Higher upfront, long-term benefit	Shokravi et al. [35]

Adverse effects and safety considerations

Pharmacological management of T2DM is associated with a range of adverse effects that vary across drug classes and therapeutic strategies, making safety a key determinant in treatment selection. Monotherapy, particularly with metformin, is generally well tolerated, with common adverse effects limited to gastrointestinal disturbances and a rare risk of lactic acidosis in predisposed individuals. However, with treatment intensification, especially involving sulfonylureas or insulin, the risks of hypoglycemia and weight gain increase.

Combination therapy, although effective in achieving rapid and sustained glycemic control, may increase the likelihood of cumulative or drug-specific adverse effects. SGLT2i are associated with genitourinary infections and volume depletion, while GLP-1 receptor agonists commonly cause gastrointestinal symptoms that may affect tolerability. The use of multiple agents may also increase regimen complexity and the potential for drug-drug interactions.

Adverse effects directly influence adherence, treatment satisfaction, and overall quality of life. Therefore, therapeutic decisions must balance efficacy with safety, emphasizing individualized treatment strategies that consider patient-specific risk factors, comorbidities, and tolerability to optimize long-term outcomes.

Personalized treatment strategies

Individualized management has become central to T2DM care due to substantial variability in disease presentation, progression, and treatment response [36]. Uniform treatment approaches are increasingly inadequate, as patient-specific factors significantly influence clinical outcomes. Stratification based on key variables such as age, duration of diabetes, baseline glycemic status, and comorbidities enables more targeted and effective interventions [4,5].

For example, younger patients with early-stage disease may benefit from intensive strategies aimed at early glycemic control and preservation of β-cell function [37]. In contrast, older individuals or those with long-standing disease often require personalized goals prioritizing safety, minimization of hypoglycemia risk, and reduced treatment burden. Comorbid conditions, including cardiovascular disease, chronic kidney disease, and obesity, play a critical role in therapeutic selection, particularly as certain agents offer benefits beyond glycemic control [38].

Advances in precision medicine further support individualized care. Phenotype-based approaches classify patients according to dominant metabolic defects, such as insulin resistance or insulin deficiency, enabling more rational pharmacological selection [25]. Emerging evidence on genetic and metabolic biomarkers may enhance the prediction of treatment response and disease progression, thereby improving therapeutic efficiency and reducing unnecessary drug exposure [28].

Current clinical guidelines increasingly emphasize personalized treatment algorithms that incorporate comorbidities, risk of adverse effects, and patient preferences [32]. The prioritization of therapies with proven cardiovascular and renal benefits in high-risk individuals reflects a shift from glucose-centric management toward outcome-oriented care. Regional adaptations further ensure applicability across diverse healthcare settings [39].

Overall, individualized treatment strategies represent a paradigm shift toward patient-centered care, aiming to optimize efficacy, enhance safety, and improve long-term disease outcomes.

Management in pregnant women

Management of T2DM in special populations requires adaptation of standard therapeutic strategies to account for differences in physiology, comorbidities, and healthcare access [5]. In elderly patients, treatment must balance glycemic control with safety considerations. This population is at increased risk of hypoglycemia, polypharmacy, and functional decline, necessitating less stringent glycemic targets and a focus on minimizing adverse effects and preserving quality of life [7]. Agents with a lower risk of hypoglycemia, such as DPP-4 inhibitors and SGLT2i, are generally preferred, while insulin and sulfonylureas should be used cautiously [40].

Patients with established cardiovascular disease represent another high-risk group requiring targeted therapy. Given the significant contribution of cardiovascular complications to morbidity and mortality in T2DM, agents with proven cardiovascular benefits, particularly SGLT2i and GLP-1 receptor agonists, are recommended as first-line options in this subgroup [41].

In individuals with chronic kidney disease, treatment selection must consider altered pharmacokinetics and the risk of drug accumulation. Several glucose-lowering agents require dose adjustment or are contraindicated in advanced renal impairment [9]. SGLT2i have demonstrated significant renoprotective effects and are increasingly utilized, while GLP-1 receptor agonists may provide additional benefit. Careful monitoring of renal function remains essential during therapy [42].

Resource-constrained settings present additional challenges, including limited access to newer therapies and financial constraints. In such environments, treatment often relies on widely available and cost-effective agents such as metformin and sulfonylureas. While these agents remain effective, concerns regarding safety and long-term outcomes persist. Improving access to essential medications, strengthening patient education, and implementing context-specific guidelines are critical for optimizing care delivery [43].

Overall, management in special populations necessitates a personalized approach that integrates clinical risk, safety considerations, and healthcare context to achieve optimal outcomes.

Implementation in clinical practice

Translation of T2DM therapeutic strategies into routine clinical practice requires effective integration of evidence-based recommendations with real-world healthcare delivery systems [3]. Although clinical guidelines provide structured treatment algorithms, their implementation is often influenced by physician expertise, patient preferences, and systemic healthcare limitations. Differences between primary and specialist care settings further affect treatment choices, with primary care favoring simpler regimens and specialist settings more likely to adopt intensive or combination-based approaches [44].

Several barriers hinder the optimal implementation of both independent and combination therapies. Clinical inertia remains a major challenge, leading to delayed treatment intensification despite inadequate glycemic control [17]. Patient-related factors, including poor adherence, limited health literacy, and concerns regarding adverse effects, further compromise outcomes. Additionally, financial constraints and limited access to newer pharmacological agents restrict the applicability of guideline-based therapies, particularly in resource-limited settings [45].

Strategies to improve implementation focus on strengthening provider-patient interaction and adopting multidisciplinary care models. Involvement of physicians, nurses, dietitians, and diabetes educators has been shown to improve glycemic outcomes and treatment adherence [6]. Patient education and shared decision-making enhance treatment acceptance and long-term compliance by aligning therapy with individual preferences. Furthermore, integration of digital health technologies, including continuous glucose monitoring and telemedicine, offers opportunities for real-time monitoring and timely treatment adjustments [46].

Overall, effective implementation of T2DM management requires a coordinated, patient-centered approach that addresses clinical, behavioral, and system-level barriers. The key factors influencing the real-world application of therapeutic strategies are summarized in Table 3.

**Table 3:** Implementation factors in T2DM care T2DM: type 2 diabetes mellitus; CGM: continuous glucose monitoring

Domain	Key elements	Impact on clinical practice	Reference
Guideline translation	Evidence-based algorithms, clinical protocols	Variability in adoption due to physician experience and healthcare infrastructure differences	Aristizábal-Colorado et al. [34]
Healthcare setting differences	Primary care vs. specialist care	Primary care favors simpler regimens; specialists adopt intensive or combination therapies	Chamberlain et al. [39]
Clinical inertia	Delay in treatment intensification	Prolonged hyperglycemia and increased complication risk	Atun et al. [40]
Patient-related factors	Adherence, health literacy, perception of side effects	Reduced treatment effectiveness and poor glycemic control	Reach et al. [42]
Economic constraints	Cost of medications, insurance coverage	Limited access to newer agents and guideline-based therapies	Romera et al. [45]
Access to therapies	Availability of advanced pharmacological agents	Disparities in treatment outcomes, especially in low-resource settings	Zaccardi et al. [9]
Multidisciplinary care	Involvement of the healthcare team (physicians, nurses, dietitians)	Improved glycemic outcomes and adherence	Okemah et al. [43]
Patient engagement	Education, shared decision-making	Enhanced compliance and long-term disease management	Atun et al. [40]
Digital health integration	CGM, telemedicine, remote monitoring	Improved real-time monitoring and timely treatment adjustments	Pan et al. [16]

Limitations and Future Directions

This review highlights several gaps in the current understanding and application of therapeutic strategies in T2DM. Much of the existing evidence is derived from randomized controlled trials involving selected patient populations, which may not fully reflect real-world clinical practice. In addition, there is limited comparative evidence on long-term outcomes, safety, and patient-centered measures between independent and combination therapeutic approaches. Variability in adherence, healthcare access, and socioeconomic factors further complicates the generalizability and implementation of these strategies.

Future research should prioritize the generation of robust real-world evidence to support more context-relevant clinical decision-making across diverse populations. Comparative studies evaluating early combination therapy vs. conventional stepwise approaches are essential to define optimal treatment pathways. Advances in precision medicine, including the identification of predictive genetic and metabolic biomarkers, may enable more targeted and effective individualized therapy. Furthermore, integration of digital health technologies with patient-centered care models is expected to enhance long-term disease management, improve adherence, and optimize clinical outcomes.

## Conclusions

This review underscores that optimal management of T2DM requires a balanced and individualized approach that integrates both independent (stepwise) and combination therapeutic strategies. Independent approaches remain practical and cost-effective, particularly in early disease stages and resource-limited settings; however, their limitations in addressing the multifactorial nature of T2DM and achieving timely glycemic control are evident. In contrast, combination therapies provide a more comprehensive strategy by simultaneously targeting multiple pathophysiological pathways, resulting in faster and more durable glycemic control. These approaches also offer additional cardiovascular and renal benefits in selected high-risk populations. Nevertheless, their use must be carefully balanced against considerations of cost, treatment complexity, accessibility, and potential adverse effects. Accordingly, therapeutic decision-making should be guided by a multidimensional framework that incorporates disease severity, underlying pathophysiology, comorbidities, safety profile, economic factors, and patient preferences. This approach reflects a shift toward personalized, evidence-based, and patient-centered care. Future progress in T2DM management will depend on integrating real-world evidence, advancing precision medicine, and adopting context-specific treatment models. Such developments are expected to enhance clinical outcomes, improve quality of life, and support more rational and efficient decision-making in routine practice.
